# Optimized formulation of a three-component extract mixture from Moroccan *Crocus sativus* L. (Stigmas, leaves, and Tepals) for enhanced antioxidant activity

**DOI:** 10.1186/s40643-025-00892-7

**Published:** 2025-09-01

**Authors:** Abdellah Baraich, Amine Elbouzidi, Mohamed Jeddi, Mohamed Taibi, Mounir Haddou, Naoufal El Hachlafi, Reda Bellaouchi, Ramzi A. Mothana, Mohammed F. Hawwal, Mohamed Addi, Larbi Rhazi, Redouane Benabbes, Abdeslam Asehraou, Bassem Jaouadi, Ennouamane Saalaoui

**Affiliations:** 1 Laboratoire de Bioressources, Biotechnologie, Ethnopharmacologie et Santé (LBBES), Faculté des Sciences d’Oujda (FSO), Université Mohammed Premier (UMP), Bd Mohamed VI BP 717, Oujda, 60000 Morocco; 2 Laboratoire d’Amélioration des Productions Agricoles, Biotechnologie et Environnement (LAPABE), Faculté des Sciences, Université Mohammed Premier, Oujda, 60000 Morocco; 3 Laboratory of Microbial Biotechnology and Bioactive Molecules, Faculty of Sciences and Technologies Faculty, Sidi Mohamed Ben Abdellah University, P.O. Box 2202, Imouzzer Road, Fez, Morocco; 4 Faculty of Medicine and Pharmacy, Ibn Zohr University, Guelmim, 81000 Morocco; 5 Department of Pharmacognosy, College of Pharmacy, King Saud University, Riyadh, 11451 Saudi Arabia; 6 Institut Polytechnique UniLaSalle, Université d’Artois, ULR 7519, 19 rue Pierre Waguet, BP 30313, Beauvais, 60026 France; 7 Laboratoire des Biotechnologies Microbiennes et Enzymatiques et de Biomolécules (LBMEB), Centre de Biotechnologie de Sfax (CBS), Université de Sfax (USF), Route Sidi Mansour Km 6, B.P. 1177, Sfax, 3018 Tunisia

**Keywords:** *Crocus sativus* L., By-products, Experimental mixture design, Antioxidant activity, DPPH, ABTS, Food preservation, Biopharmaceuticals

## Abstract

This study evaluated the antioxidant potential of a three-component mixture derived from *Crocus sativus L.* stigmas, leaves, and tepals, aiming to valorize these underutilized by-products. Hydroethanolic extracts were chemically profiled using High-Performance Liquid Chromatography with Diode-Array Detection (HPLC-DAD), identifying major bioactive compounds including crocin (11.04%), rutin (22.32%), and ellagic acid (62.47%). Antioxidant capacity was assessed using DPPH and ABTS assays, yielding IC_50_ values ranging from 88.67 ± 0.83 *µ*g/mL to 291.41 ± 0.91 *µ*g/mL for DPPH, and from 116.76 ± 1.31 *µ*g/mL to 286.87 ± 0.25 *µ*g/mL for ABTS. A simplex-centroid mixture design was employed to determine optimal component ratios. The best-performing DPPH mixture (43% stigmas, 35% leaves, 22% tepals) achieved an IC_50_ of 97.36 ± 1.23 *µ*g/mL, closely matching the predicted 95.96 *µ*g/mL. Similarly, the optimal ABTS blend (45% stigmas, 35% leaves, 20% tepals) reached an IC_50_ of 110.59 ± 6.82 *µ*g/mL, aligning with the predicted 106.31 *µ*g/mL. Both combinations outperformed standard antioxidants like BHT and ascorbic acid. These findings support the synergistic use of saffron by-products in food preservation, nutraceuticals, and biopharmaceuticals, offering an eco-friendly, cost-effective solution aligned with circular economy principles.

**Supplementary Information:**

The online version contains supplementary material available at 10.1186/s40643-025-00892-7.

## Graphical Abstract

**Supplementary Information:**

The online version contains supplementary material available at 10.1186/s40643-025-00892-7.

## Graphical abstract

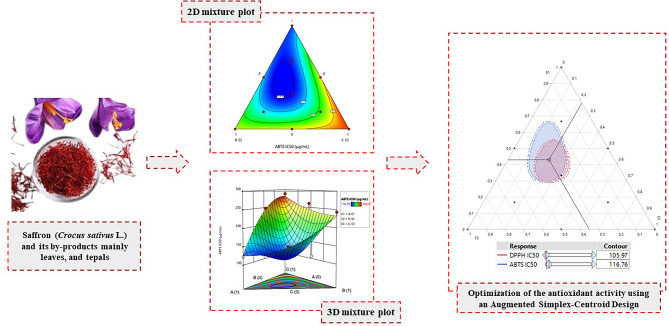

## Introduction

*Crocus sativus* L.), often referred to as “red gold,” is one of the most valuable and sought-after spices worldwide. Primarily cultivated for its vivid red stigmas, saffron has been prized for centuries for its wide-ranging applications in gastronomy, traditional medicine, cosmetics, and pharmacology. Iran, the global leader in production, accounts for 80–90% of the market, with over 300 tons produced annually (Hosseinzadeh et al., 2013; Tarantilis et al. 1995). However, other regions also contribute significantly to saffron cultivation, including Morocco, where the Taliouine region, known as the “saffron capital,” produces approximately 6 tons per year, representing nearly 7% of global production (Khorasany et al., 2016; Moratalla-López et al. 2019). This production is furtherenhanced by the recognition of Geographical Indications (GI), which elevate the profile of Moroccan saffron in international markets.

Despite the high demand for saffron stigmas, its cultivation generates a substantial number of by-products, including tepals and leaves, which constitute up to 90% of the flower’s biomass. These by-products are often overlooked, discarded, or used as compost, despite their richness in bioactive compounds. Tepals, for instance, represent approximately 930 kg per ton of harvested flowers and are rich in flavonoids, anthocyanins, and phenolic acids, which are recognized for their antioxidants, anti-inflammatory, and anti-aging properties (Rezaee et al., 2013). Leaves, though less studied, also contain flavonoids, polyphenols, and essential micronutrients, offering potential applications in dietary supplements or as biostimulants (Wu et al. 2023).

These by-products represent an underutilized potential for nutraceutical, pharmaceutical, and cosmetic industries. Recent research suggests that formulations combining stigmas, tepals, and leaves may exhibit synergistic effects, particularly in enhancing antioxidant activity. This activity is crucial in mitigating oxidative stress, a key factor in the development of chronic diseases such as cardiovascular diseases, diabetes, and neurodegenerative disorders (Rahmouni et al. 2017; Yuan et al. 2020; Zhu et al. 2021). Incorporating these by-products into a circular value chain could not only reduce agricultural waste but also improve profitability for producers, particularly in regions like Taliouine.

This study aims to systematically explore the synergistic interactions among the stigmas, tepals, and leaves of *Crocus sativus* L., and to optimize their antioxidant activities using DPPH and ABTS assays. More specifically, the objectives are to determine the optimal proportions of these three components to maximize antioxidant efficacy while minimizing the quantities required, assess the valorization potential of these by-products, which are often regarded as agricultural waste, and propose a practical and economically viable solution to enhance the profitability of producers, particularly in the Taliouine region. To achieve these goals, we employed a simplex-centroid design, a rigorous statistical method that enables the systematic evaluation of various combinations. This work is distinguished by its innovative approach applied to saffron, a species of significant cultural and economic importance worldwide. While the pharmacological properties of the stigmas are well-documented, the synergistic potential of the by-products (tepals and leaves) in combination with stigmas remains largely unexplored. These by-products, often discarded or used as animal feed, contain promising bioactive compounds. By investigating these interactions, this research paves the way for a sustainable and efficient utilization of saffron by-products, thereby contributing to a circular value chain and the overall optimization of saffron production.

## Materials and methods

### Plant material and extraction of extracts

*Crocus sativus* L., commonly known as saffron, was collected from the Taliouine region in southern Morocco (30°31′54″ N, 7°55′25″ W). The botanical identification was conducted by Professor Fennane Mohammed, a renowned botanist at the Institut Scientifique in Rabat, Morocco. The sample has been cataloged under the herbarium code HUMPOM210 within the collection of the Mohammed Premier University in Oujda. The chemical and structural composition of *Crocus sativus* L. can vary significantly based on several factors, including the variety of *Crocus sativus* L., environmental growth conditions, and cultivation techniques used. In terms of proportion, the stigmas generally represent a minimal fraction of the dried saffron’s total weight, roughly 0.5 to 1%, while the tepals and leaves constitute the majority.

The preparation of plant material followed the method described by Ouahhoud et *al*. (Ouahhoud et al. 2023). The different parts of *Crocus sativus* L. (stigmas, tepals, and leaves) were manually separated before being dried in an oven (JP SELECTA, Spain) at 37 °C: stigmas for 4 h, and tepals and leaves for 24 h. Subsequently, the dried materials underwent maceration in a hydroethanolic mixture (ethanol/water, 80/20, v/v) for 24 h at room temperature, with constant agitation (Witeg MS-MP8, Germany) and in darkness. An extraction ratio of 2 g of plant powder per 50 mL of solvent was applied. After each extraction, the solution was filtered using a syringe filter (0.45 *µ*m), and the residual marc was reused for additional extractions. The combined hydroethanolic phases were concentrated at 40 °C using a rotary evaporator (Buchi B-480, Switzerland), and the dry extracts (stigmas, tepals, and leaves) were stored at − 20 °C in a freezer (BOSCH, Germany). The extraction yields, expressed as a percentage relative to the dry material, were 62.15% for stigmas, 63.77%for tepals, and 25.3% for leaves (Eq. 1). hese variations reflect the intrinsic differences in the biochemical composition of the various plant parts.

1 $$\eqalign{& {\bf{Extraction}}{\mkern 1mu} {\bf{yield}}{\mkern 1mu} \left( \% \right) \cr & = \left[ {\left( {{{{\bf{Weight}}{\mkern 1mu} {\bf{of}}{\mkern 1mu} {\bf{extracted}}{\mkern 1mu} {\bf{compounds}}} \over {{\bf{Weight}}{\mkern 1mu} {\bf{of}}{\mkern 1mu} {\bf{dry}}{\mkern 1mu} {\bf{plant}}{\mkern 1mu} {\bf{materia}}}}} \right) \times 100} \right] \cr} $$

### HPLC-DAD analysis

The chromatographic analysis was conducted using a high-performance liquid chromatography (HPLC) system equipped with a diode-array detector (DAD). Analyte separation was performed on a ZORBAX Eclipse XDB-C18 column (Agilent) with dimensions of 4.6 × 250 mm and a particle size of 5 *µ*m. The column temperature was maintained at 40 °C to ensure consistent separation efficiency and reproducibility.

The mobile phase consisted of two solvents: water (solvent A) and acetonitrile (solvent B). Gradient elution was employed with the following program: starting at 10% solvent B, increasing linearly to 50% at 22 min, and further to 100% at 32 min. This composition was maintained until 40 min before being reduced back to 10% at 44 min. A re-equilibration phase was included, resulting in a total run time of 50 min. The mobile phase flow rate was consistently maintained at 0.5 mL/min throughout the analysis.

Detection was carried out using DAD, with chromatograms recorded at three wavelengths: 254 nm, 280 nm, and 330 nm. The study utilized a range of analytical standards, including apigenin, caffeic acid, chlorogenic acid, crocin, ellagic acid, epicatechin, gallic acid, ferulic acid, luteolin, *p*-coumaric acid, protocatechuic acid, pyrocatechol, quercetin, rutin, and vanillic acid. These standards were used to calibrate the system, enabling precise identification and quantification of the target compounds. Calibration curves were constructed for each standard using known concentrations, and method validation was conducted to ensure linearity, precision, and accuracy.

### Antioxidant assays

#### DPPH radical scavenging assay

The antiradical potential of three individual extracts and their various combinations, formulated through an experimental design methodology, was evaluated using the 2,2-diphenyl-1-picrylhydrazyl (DPPH) assay. This assessment employed a modified protocol adapted from Elbouzidi et al. (2024) (Elbouzidi et al. 2024).

#### ABTS radical scavenging activity

The ABTS radical scavenging activity, the procedure was carried out according to the methods described by Al-Mijalli et *al*. (Al-Mijalli et al. 2023) and El Hachlafi et *al*. (El Hachlafi et al. 2024), with minor adjustments.

### Experimental design

#### Mixture design

The simplex-centroid design incorporated vertex points (pure components), edge midpoints (binary mixtures), and a design center (ternary mixture) to systematically evaluate linear and interaction effects. Supplementary check points (67:16:17 ratios) were included to refine model accuracy in critical regions. This optimized design enables efficient exploration of synergistic effects while minimizing experimental runs, consistent with established mixture design methodology (Benkhaira et al. 2023). The composition of the CSE mixture, outlined in Table 1, allows each component to vary between 0 and 1, with their total always equaling 1. The antioxidant activity of the CSE was measured using the DPPH IC_50_ and ABTS IC_50_ parameters.

**Table 1:** Identification of independent variables used in the mixture

Components	Coded variables	Level -	Level +
CSSE (E1)	X_1_	0	1
CSLE (E2)	X_2_	0	1
CSTE (E3)	X_3_	0	1
**Sum of proportions**		**1**	

#### Experimental matrix and mathematical model

**Fig. 1:**
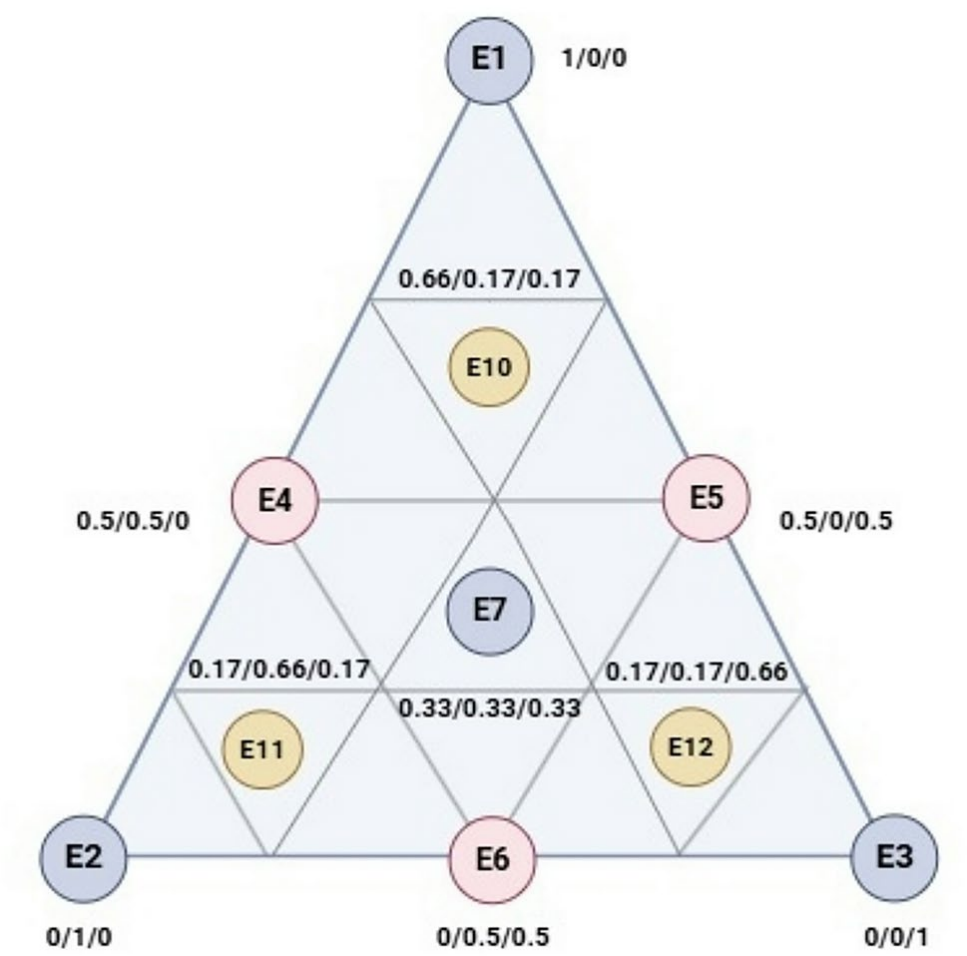
Equilateral triangle of the arrangement of mixtures using the simplex centroid design method. E1: *Crocus sativus* stigmas extract (CSSE); E2: *Crocus sativus* Leaves extract (CSLE); E3: *Crocus sativus* tepals extract (CSTE). E4-10: experimental combinations

In this study, 10 experimental trials were designed and visualized within an equilateral triangle (Fig. 1). The triangle vertices (X_1_, X_2_, and X_3_) represent the three pure components (1), while the midpoints (X_4_, X_5_, and X_6_) correspond to binary combinations (0.5/0.5). The centroid of the triangle (X_7_) represents an equal proportion of all three components (0.33/0.33/0.33). Each trial was performed in triplicate, and three additional control points (X_10_, X_11_, and X_12_) were included to represent ternary mixtures with proportions of 0.67/0.16/0.16. A cubic model was employed to describe the responses in relation to the independent variables, as expressed in the following equation (Eq. 2):

2 $$\eqalign{{{Y}} = & {{{\theta }}_1}{{{X}}_1} + {{{\theta }}_2}{{{X}}_2} + {{{\theta }}_3}{{{X}}_3} + {{{\theta }}_{12}}{{{X}}_1}{{{X}}_2} \cr & + {{{\theta }}_{13}}{{{X}}_1}{{{X}}_3} + {{{\theta }}_{23}}{{{X}}_2}{{{X}}_3} + {{{\theta }}_{123}}{{{X}}_1}{{{X}}_2}{{{X}}_3} + {\rm{\varepsilon }} \cr} $$

In this context, Y represents the experimental response measured as IC_50_ (*µ*g/mL). The symbols θ_1_, θ_2_, and θ_3_ denote the linear regression coefficients, while θ_12_, θ_13_, and θ_23_ represent the interaction coefficients for pairs of variables. The coefficient θ_123_ accounts for the ternary interactions, and ɛ signifies the error term in the regression model.

### Statistical analysis

To assess the statistical significance of the mathematical model at a 95% confidence level, we utilized the F_ratio (MSR/MSr)_ between the mean square regression and the mean square residual. A higher Fratio value indicates greater variability of the results around their mean. Additionally, we examined the ratio of the mean square Lack of Fit (MSLOF) to the mean square Pure Error (MSPE), referred to as FratioLOF/PE. This ratio is used to evaluate the adequacy of the proposed mathematical model by comparing the model’s lack of fit to the inherent variability in the data. High FratioLOF/PE values suggest that the model may not adequately describe the observed data, indicating a poor fit.

The coefficient of determination (R²) was calculated to evaluate the quality of the mathematical models. The t-test was employed to assess the significance of the estimated factors, while the ANOVA F-test was conducted to confirm the significance of the models. The experimental design, along with graphical and statistical analyses, were performed using Design Expert software version 12 and SAS JMP^®^ version 14. Results are presented as means ± standard deviation (SD), with *n* = 3.

## Results

### Chemical profile of the three extracts

The Table 2 provides a detailed analysis of the chemical compounds identified in extracts from different parts of *Crocus sativus* L. (stigmas, leaves, and tepals) obtained via EtOH/H₂O extraction. The compounds were characterized by their retention times (RT), corresponding standards, and relative concentrations expressed as percentage areas (% Area). This analysis highlights the diversity of secondary metabolites, including phenolic acids, flavonoids, and carotenoids.

The HPLC-DAD analysis provided a comprehensive phytochemical characterization of the different saffron extracts (Supplementary Figures S1-S3, table S1). Quantitative analysis revealed distinct compositional profiles for each plant part. For the leaf extracts, ellagic acid emerged as the predominant compound, representing 62.47 ± 0.52% of the total identified constituents. This was followed by crocin (1.33 ± 0.05%) and caffeic acid (1.28 ± 0.03%) as secondary components. Minor phenolic compounds included gallic acid (0.87 ± 0.02%) and protocatechuic acid (0.23 ± 0.01%). The cumulative percentage of characterized compounds reached 67.83% of the total extract composition. The stigma extracts displayed a different phytochemical pattern, with crocin (11.0392 ± 0.41%) and ellagic acid (6.0039 ± 0.23%) as the most abundant compounds. Significant quantities of apigenin (5.7777 ± 0.15%) were also detected. The total identified compounds accounted for 30.006% of the stigma extract composition. Tepal extracts showed yet another distinct profile, dominated by ellagic acid (50.48 ± 0.47%) and rutin (22.32 ± 0.39%). While crocin was present at 11.04 ± 0.21%, various phenolic acids collectively contributed to the remaining composition. The overall characterization rate for tepal extracts reached 30.01% of total constituents.

Finally, common molecules such as crocin and rutin are present across all fractions, although their proportions vary depending on the part analyzed. These results underline the chemical complementarity of the different extracts and their potential for diverse pharmacological and cosmetic applications.

**Table 2:** Phytochemical profile of the extracts using HPLC-DAD

Plant Part	Dominant Compounds (> 1%)	Relative abundance (% Area)	Total Identified
Leaves	**Ellagic acid**	**62.4659**	67.8316
	Crocin	1.3277	
	Caffeic acid	1.2780	
	Other phenolic compounds	2.75	
Stigmas	**Crocin**	**11.0392**	30.006
	Ellagic acid	6.0039	
	Apigenin	5.7777	
	Quercetin	2.8638	
	Rutin	2.0428	
	Other phenolic compounds	2.2793	
Tepals	**Ellagic acid**	**50.4781**	80.7258
	Rutin	22.3230	
	Pyrocatechol	3.2820	
	Ferulic acid	2.6208	
	Other phenolic compounds	2.0219	

RT: retention time; mAU*s: milli-Absorbance Units* seconds

### Antioxidant activity of individual extracts

The presented bar graphs illustrate the antioxidant activities of various extracts of *Crocus sativus* (CSSE, CSLE, CSTE) compared to standard antioxidants BHT (butylated hydroxytoluene) and AA (ascorbic acid) using DPPH and ABTS assays. The stigma extract (CSSE) demonstrated the strongest antioxidant activity in both DPPH and ABTS assays, evidenced by the lowest IC₅₀ values among the extracts. This high efficiency is attributed to the abundance of carotenoids, such as crocin and crocetin, along with phenolic acids like ellagic acid and flavonoids such as quercetin and rutin. The stigma extract (CSSE) exhibited the highest antioxidant potential among the individual extracts in both DPPH and ABTS assays, consistent with its high content of carotenoids (e.g., crocin) and polyphenols (e.g., rutin and quercetin), followed by leaf and tepal extracts. The potent antioxidant activity of CSSE supports its traditional use in pharmacological applications and highlights its therapeutic potential in managing oxidative stress.

In the leaves, ellagic acid stands out as the dominant compound, accounting for 62.47% of the total identified metabolites. This finding highlights its significant role as a major antioxidant, consistent with the work of Verotta et al. (2018) (Verotta et al. 2018), who emphasized its potent antioxidant and anti-inflammatory properties. In addition to ellagic acid, crocin (1.33%) and caffeic acid (0.28%) contribute to the ioactive profile of the leaves. Phenolic molecules such as gallic acid and protocatechuic acid, although detected in smaller proportions, are also noteworthy. These phenolic compounds are known for their biological activities, including anti-inflammatory, antimicrobial, and neuroprotective effects, as reported by Rafie et al. (2017) (Rafie et al. 2017). These findings confirm the bioactive potential of *C. sativus* leaf extracts, suggesting their possible application in pharmacological and nutraceutical fields.

Tepals, often considered a by-product, have demonstrated significant antioxidant activity in the ABTS assay, although this is weaker than that observed in CSLE. This reduced activity can be attributed to the high levels of rutin (22.32%) and ellagic acid (50.48%) present, as reported in previous studies (Han et al. 2006; Nguyen et al. 2019; Rusmana et al. 2017). Furthermore, their activity in the DPPH assay was also found to be lower than that of CSSE. Nevertheless, tepals hold promising potential for nutraceutical applications due to their rich profile of flavonoids and phenolic acids. Utilizing them could enhance the economic value of *Crocus sativus* cultivation by transforming this residual material into formulations rich in antioxidants. Synthetic antioxidants such as butylated hydroxytoluene (BHT) and natural antioxidants like ascorbic acid (AA) exhibited superior radical scavenging activities compared to all *Crocus sativus* extracts, as evidenced by their lower IC₅₀ values in both assays. These findings underscore the benchmark efficacy of commercial antioxidants. However, the considerable activity of natural extracts, particularly stigmas and tepals, highlights their potential as safer, plant-based alternatives to synthetic antioxidants.

**Fig. 2:**
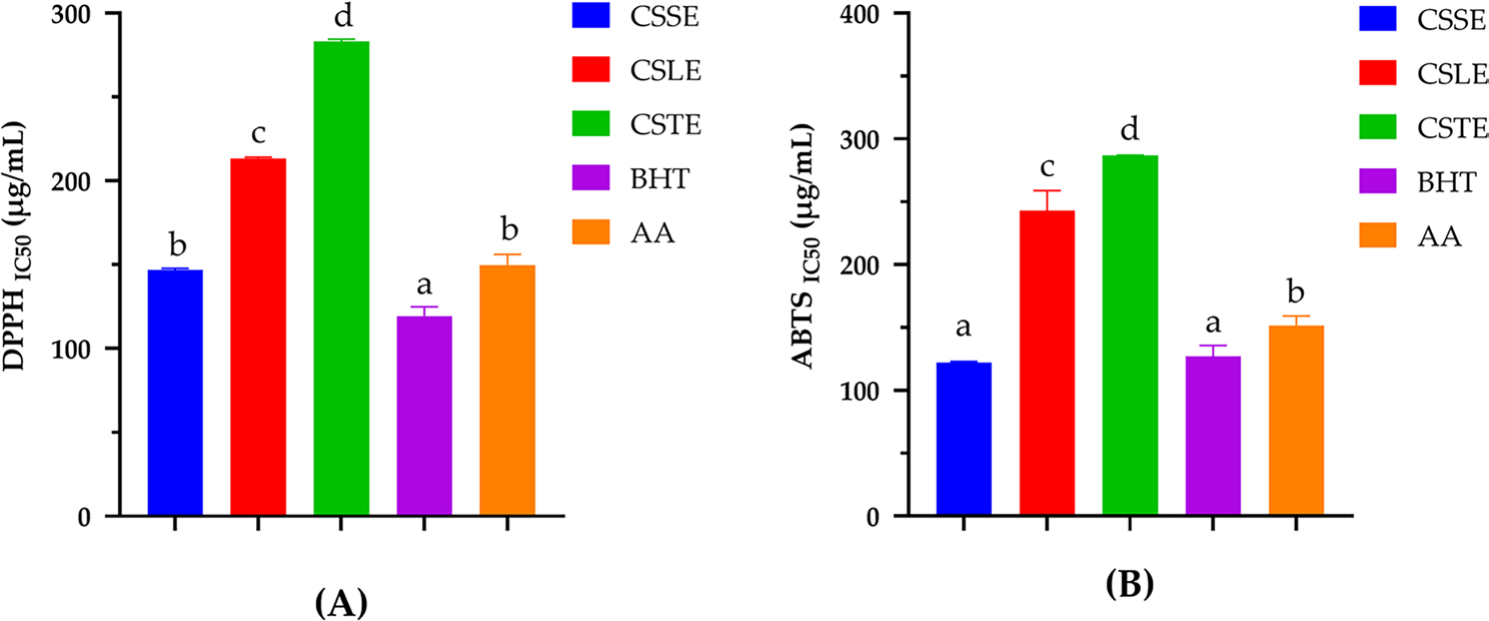
Antioxidant activity of the studied extracts, through DPPH assay (**A**), and ABTS test (**B**). butylated hydroxytoluene (BHT) and ascorbic acid (AA) were used as standards. Data presented as mean ± SD of three independent experiments. Different letters indicate a statistically significant difference between the groups at *p* < 0.05

This study demonstrates that the antioxidant capacity of *Crocus sativus* varies significantly among its parts. Stigmas exhibits the highest activity, aligning with their established role in pharmacological use. Leaves, traditionally discarded, show promising potential due to their rich content of bioactive compounds. Tepals, while less potent, still contribute to the plant’s overall antioxidant profile. These findings suggest that all parts of *Crocus sativus* could be valorized for nutraceutical and therapeutic applications, minimizing waste and maximizing the plant’s economic and biological value (Fig 2).

### Simplex centroid design

Table 3 outlines the simplex-centroid experimental design, which examines various combinations of three *Crocus sativus* L. extracts and evaluates their antioxidant effects using DPPH IC_50_ and ABTS IC_50_ indices. These extracts, widely recognized for their health-promoting properties, hold significant potential for the development of products aimed at reducing oxidative stress while providing additional therapeutic benefits. To date, no published study has investigated the combined effects of the extracts derived from stigmas, leaves, and tepals using this specific methodology, making this research both innovative and noteworthy.

The study involved 12 randomized trials, with each result representing the average of three independent tests [56]. Antioxidant activity ranged from 88.67 ± 0.83 to 291.41 ± 0.91 *µ*g/mL for DPPH IC_50_ and from 116.76 ± 1.31 to 286.87 ± 0.25 *µ*g/mL for ABTS IC_50_. Among the combinations tested, mixture number 7, comprising equal proportions of CSSE, CSLE, and CSTE (0.33, 0.33, and 0.33), showed the best performance in the DPPH assay, whereas mixture number 11, with proportions of 0.17, 0.67, and 0.17, respectively, was the most effective in the ABTS assay. The ternary mixtures showed statistically significant improvements in antioxidant activity compared to standard references, namely butylated hydroxytoluene (123.43 ± 6.44 *µ*g/mL for DPPH and 168.22 ± 10.23 µg/mL for ABTS) and ascorbic acid (147.81 ± 5.33 *µ*g/mL for DPPH and 140.22 ± 8.99 *µ*g/mL for ABTS). Both mixtures achieved the lowest IC_50_ values in the DPPH and ABTS tests, highlighting their potent antioxidant potential.

**Table 3:** Matrix of simplex centroid design and results for DPPH IC_50_, and ABTS IC_50_

No. ^a^	CSSE (E1)	CSLE (E2)	CSTE (E3)	Observed responses ^b^ (µg/mL)	
				DPPH IC_50_	ABTS IC_50_
1	1	0	0	146.81 ± 1.06	122.29 ± 0.71
2	0	1	0	213.32 ± 0.85	242.98 ± 15.87
3	0	0	1	283.33 ± 1.15	286.87 ± 0.25
4	0.5	0.5	0	176.44 ± 0.22	154.6 ± 0.98
5	0.5	0	0.5	254.65 ± 1.02	247.83 ± 6.12
6	0	0.5	0.5	291.41 ± 0.91	261.45 ± 0.87
7	0.33	0.33	0.33	105.97 ± 0.09	139.32 ± 2.13
8	0.33	0.33	0.33	125.92 ± 0.06	125.56 ± 2.55
9	0.33	0.33	0.33	115.98 ± 0.12	129.34 ± 2.44
10	0.67	0.17	0.17	117.19 ± 0.32	123.72 ± 0.48
11	0.17	0.67	0.17	133.81 ± 0.47	116.76 ± 1.31
12	0.17	0.17	0.67	165.53 ± 0.91	172.36 ± 2.48
BHT *	-	-	-	119.43 ± 5.44	127.15 ± 8.70
AA**	-	-	-	149.81 ± 6.33	151.61 ± 7.45

^a^ Experiments were performed after randomization. ^b^ The tests were conducted in three independent replicates and established as means ± SD. * BHT: Butylated hydroxytoluene; ** AA: Ascorbic acid

### Statistical validation of postulated model

**Fig. 3:**
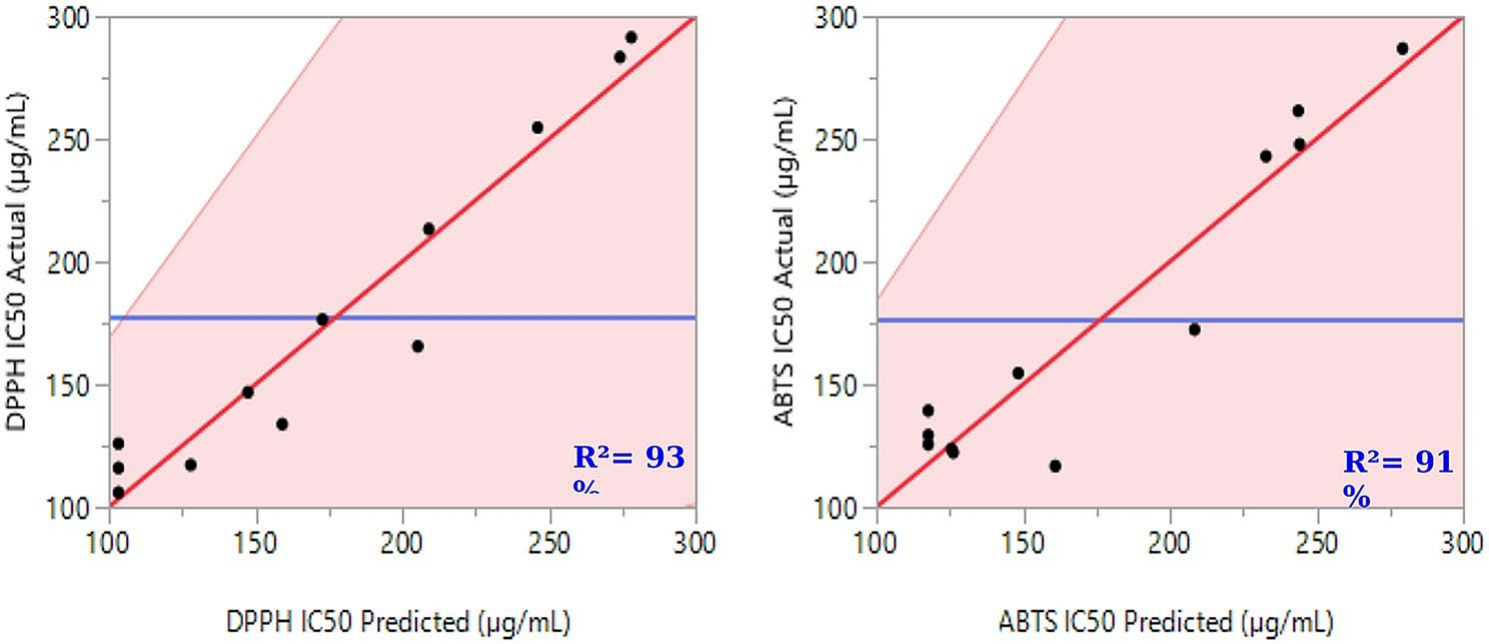
The red lines represent the trend of the experimental values compared to the predicted values for both responses, DPPH IC_50_ and ABTS IC_50_. Meanwhile, the blue lines indicate the observed mean values for the two analyzed responses

An analysis of variance (ANOVA) was conducted to examine the interactions among the components of the mixture, as shown in Table 4. The results indicated that the main effects of the regression model were statistically significant for both responses (DPPH IC_50_ and ABTS IC_50_), with *p*-values below 0.05 (0.0087 and 0.0212, respectively). The calculated F-values (11.3801 for DPPH IC_50_ and 7.5403 for ABTS IC_50_) exceeded the critical F-values at the 95% confidence level, confirming significant effects. Additionally, the ANOVA F-tests validated the models’ reliability, with p-values of 0.0087 and 0.0212 indicating no significant lack of fit. The F-ratios for lack of fit, compared to pure error, were below the critical value (19.16), further supporting this conclusion. High coefficients of determination (R² = 0.93 and adjusted R² = 0.84 for DPPH IC_50_; R² = 0.91 and adjusted R² = 0.78 for ABTS IC_50_) demonstrated a strong agreement between the experimental data and the model predictions. This alignment is also illustrated in Fig. 3, which shows a linear relationship between the observed and predicted values for both responses.

**Table 4:** Variance analysis for the three fitted models

Response	*R*²	Adj. *R*	F-value	*p*-value	Lack of Fit (*p*)
DPPH_IC50_	0.93	0.84	11.38	**0.0087**	0.124
ABTS_IC50_	0.91	0.78	7.54	**0.0212**	0.208

### Components effects and adjusted models

Table 4 provides a summary of the estimated regression coefficients for the specialized model. Regression equations with statistically significant coefficients (*p*-values < 0.05) were utilized to determine the relationships between the tested factors and the observed responses for DPPH IC_50_ and ABTS IC_50_ (Table 5).

**Table 5:** Coefficients of the two presumed models and their level of signifcance (*p*-value)

Term	Coefficients	DPPH_IC50_		ABTS_IC50_	
		Estimation	*p*-value	Estimation	*p*-value
E1 (Mixture)	θ_1_	147,43563	**0**,0020*	126,22596	**0**,0072*
E2(Mixture)	θ_2_	209,09018	**0**,0004*	232,8078	**0**,0005*
E3(Mixture)	θ_3_	274,27654	**0**,0001*	279,39597	**0**,0002*
E1* E2	θ_12_	-21,70848	0,8705	-124,6126	0,4305
E1* E3	θ_13_	141,46418	0,3144	165,92376	0,3054
E2* E3	θ_23_	145,77323	0,3014	-49,19273	0,7488
E1* E2* E3	θ_123_	-3684,743	**0**,0031*	-2544,428	**0**,0235*

*Statistically signifcant at *p* < 0.05

The statistically significant coefficients for the DPPH IC_50_ response are those representing the effects of individual compounds (θ_1_, θ_2_, and θ_3_), as well as the ternary interaction term θ_123_. In contrast, the coefficients for binary interaction terms (θ_12_, θ_13_, and θ_23_) are not significant (*p* > 0.05) and do not indicate any influence on the DPPH radical. After excluding all non-significant coefficients from the proposed models, the mathematical equations describing the response as a function of the tested components are expressed in Eq. (3):

3 $$\eqalign{{{Y}} = & 147,43{{{X}}_1} + 209,09{{{X}}_2} \cr & + 274,27{{{X}}_3} - 3684,743{{{X}}_1}{{{X}}_2}{{{X}}_3} + {\rm{\varepsilon }} \cr} $$

For the ABTS IC_50_ response, the significant terms were θ_1_, θ_2_, θ_3_ et θ_123_. These findings confirm that the antioxidant potential on the ABTS radical is primarily influenced by the effects of the linear terms θ_1_, θ_2_, and θ_3_, as well as the ternary interaction term. Consequently, the adopted mathematical model is expressed in Eq. (4):

4 $$\eqalign{{\rm{Y}} = & 126,22{{{X}}_1} + 232,80{{{X}}_2} \cr & + 279,39{{{X}}_3} - 2544,42{{{X}}_1}{{{X}}_2}{{{X}}_3} + {\rm{\varepsilon }} \cr} $$

### Optimization of formulation and desirability study

The optimization process, grounded in experimental design methodology, aims to determine the ideal proportions of the studied components to maximize response values. Although the optimal results predicted by statistically validated mathematical models do not always align precisely with the observed values from the 12 experimental trials, these models provide a high degree of accuracy in forecasting outcomes within the experimental framework. To identify the most favorable conditions, the process begins by targeting the highest recorded values. In this study, the best results achieved were 105.97 *µ*g/mL and 116.76 *µ*g/mL for DPPH IC_50_ and ABTS IC_50_, respectively. Conditions capable of producing response values equal to or exceeding these thresholds were deemed satisfactory.

### Mixture profile

**Fig. 4:**
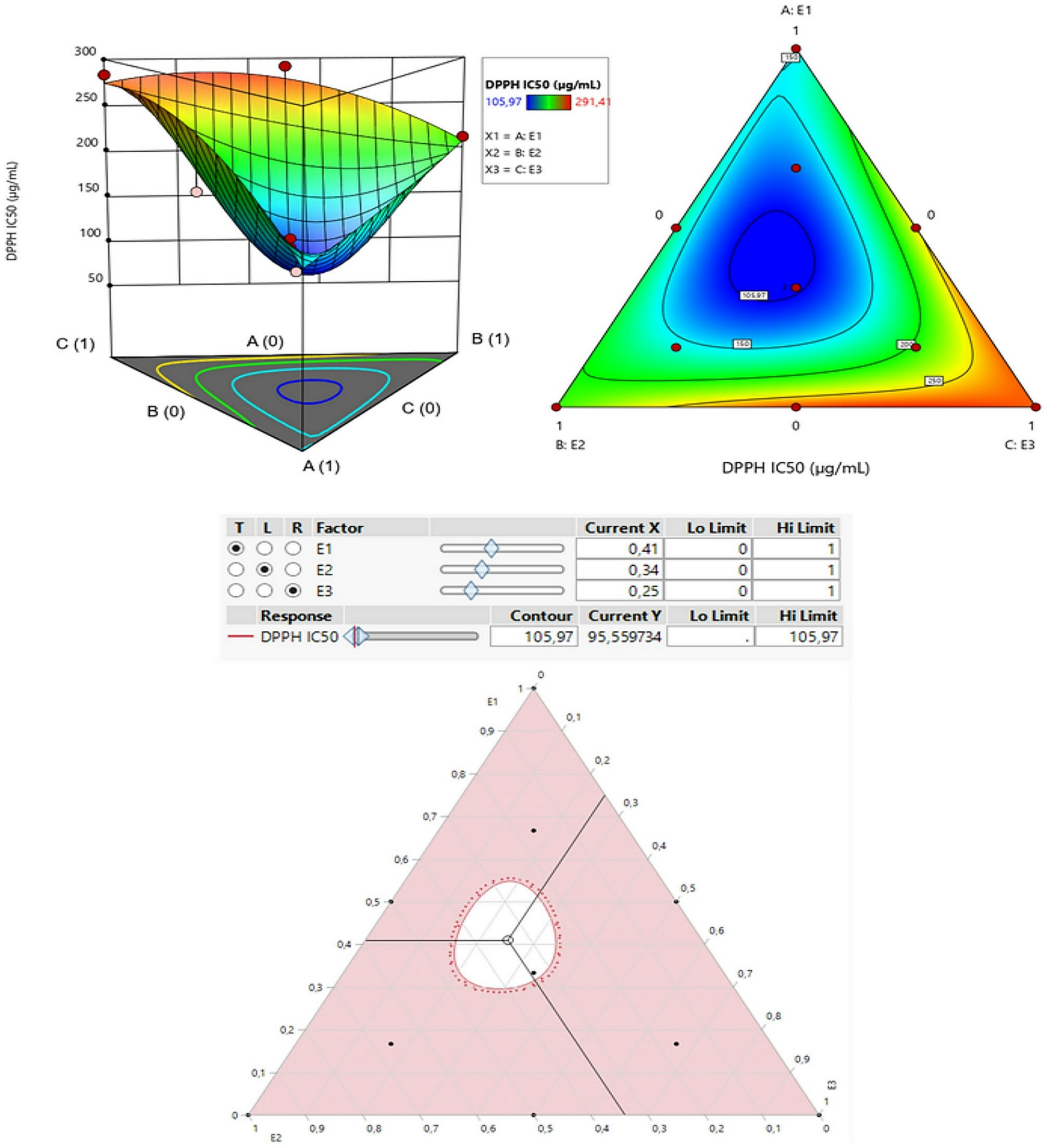
2D and 3D mixture plots of the desired compromise area, resulting in the best value of DPPH IC_50_

**Fig. 5:**
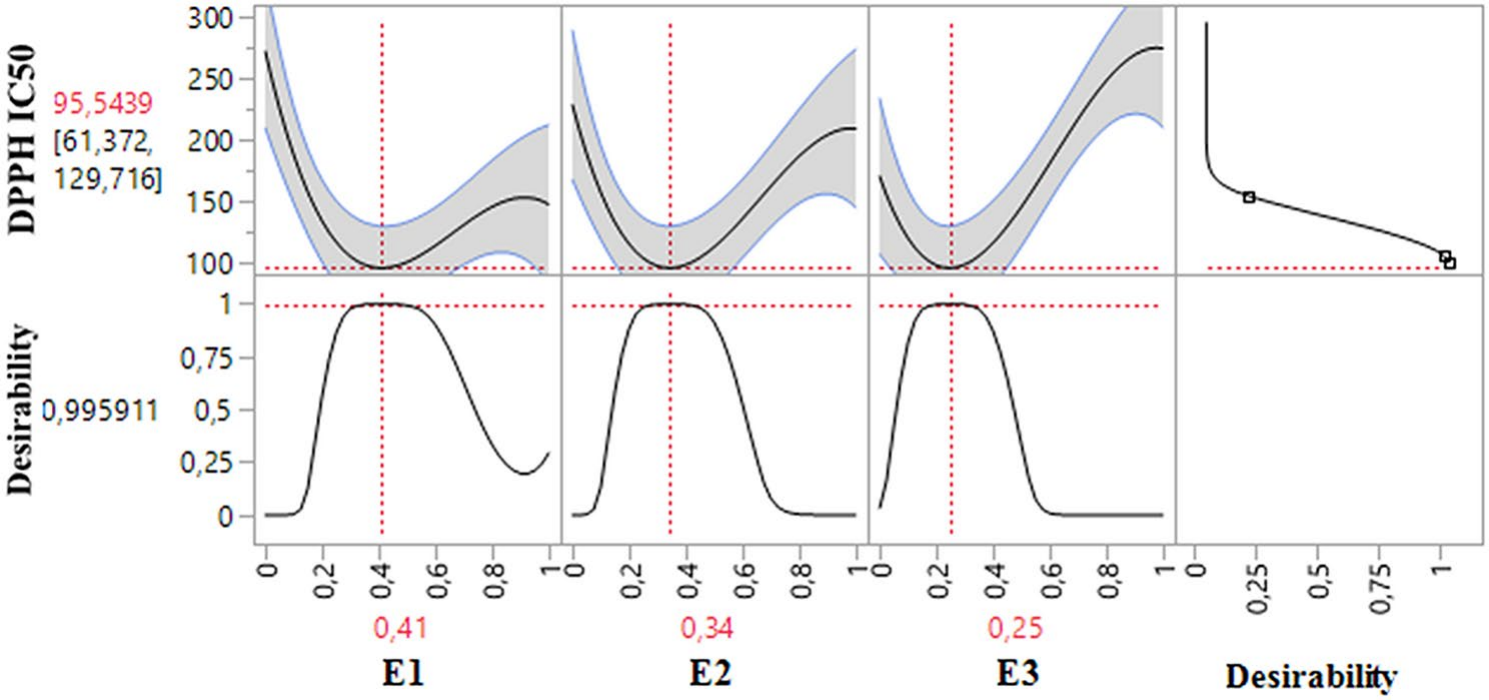
Desirability profile illustrating the precise proportions of CSSE, CSLE, and CSTE, leading to the optimum value for DPPH IC_50_

The contour plot and 3D surface graph (shown as 2D and 3D mixture plots in Figs. 4 and 5, respectively) display the optimal mix of the three extracts CSSE, CSLE, and CSTE to maximize the responses (DPPH IC_50_ and ABTS IC_50_). These visualizations highlight the relationship between the responses and the concentrations of each antioxidant. Created using Design-Expert software, these plots utilize iso-response curves, ideal for pinpointing the best conditions for achieving optimal response values. In the plots, blue indicates lower IC_50_ values and higher antioxidant effectiveness, while colors transitioning from yellow to dark red represent increasing IC_50_ values, indicating lower effectiveness.

#### Optimization of DPPH IC_50_

The 2D and 3D mixture plots (Fig. 4) reveal a dark blue region that represents the optimal compromise to achieve a DPPH IC_50_ value of 105.97 *µ*g/mL through a ternary mixture of CSSE, CSLE, and CSTE. This synergy is further validated by the desirability test results (Fig. 5), which show that a DPPH IC_50_ value of 95.54 *µ*g/mL, with a desirability score of 99%, can be obtained with the following proportions: 41% CSSE, 34% CSLE, and 25% CSTE. This antioxidant activity surpasses that of standard antioxidants such as BHT (IC_50_ = 119.43 ± 5.44 *µ*g/mL) and ascorbic acid (IC_50_ = 149.81 ± 6.33 *µ*g/mL).

#### Optimization of ABTS IC_50_

**Fig. 6:**
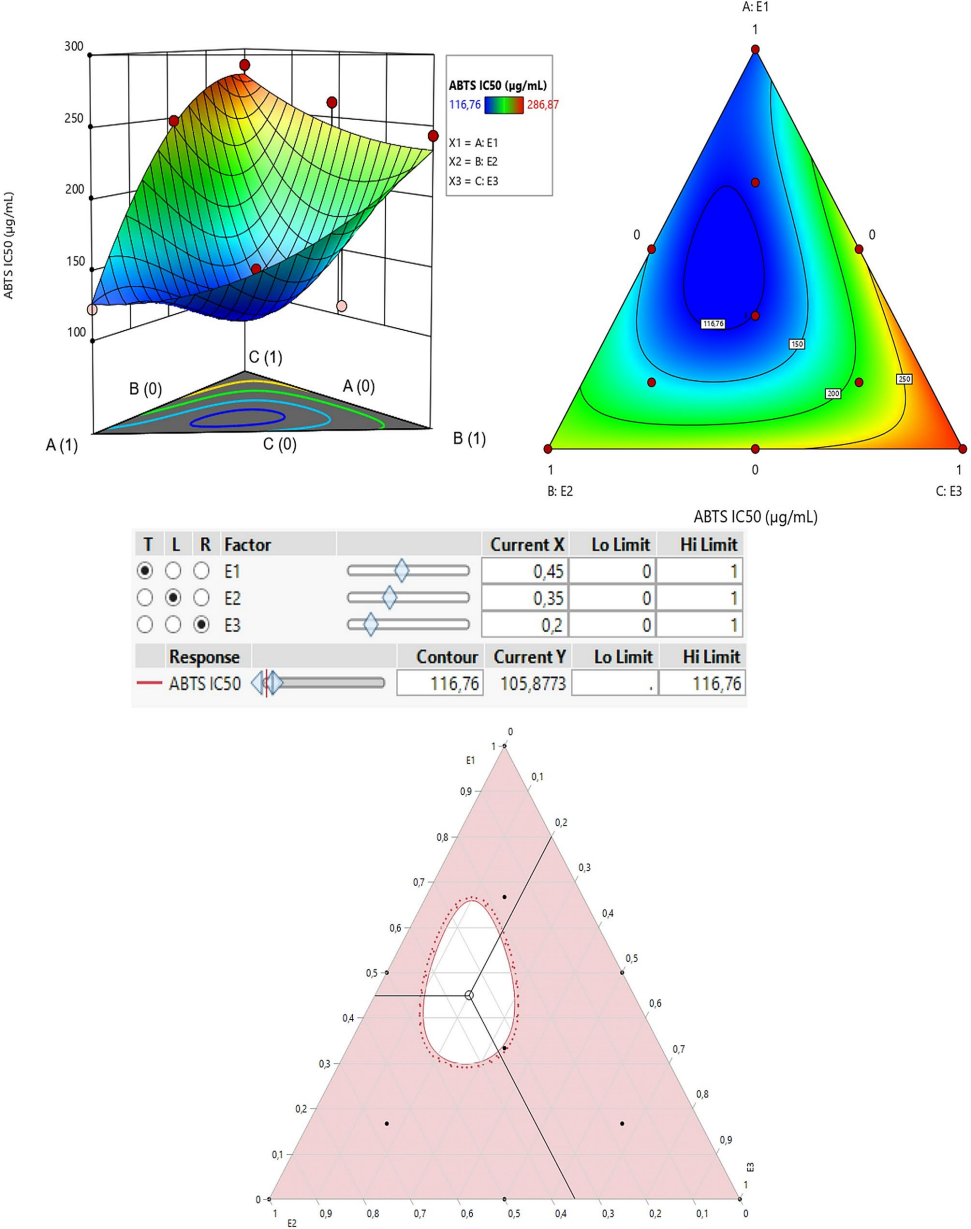
2D and 3D mixture plots of the desired compromise area, resulting in the best value of ABTS IC_50_

**Fig. 7:**
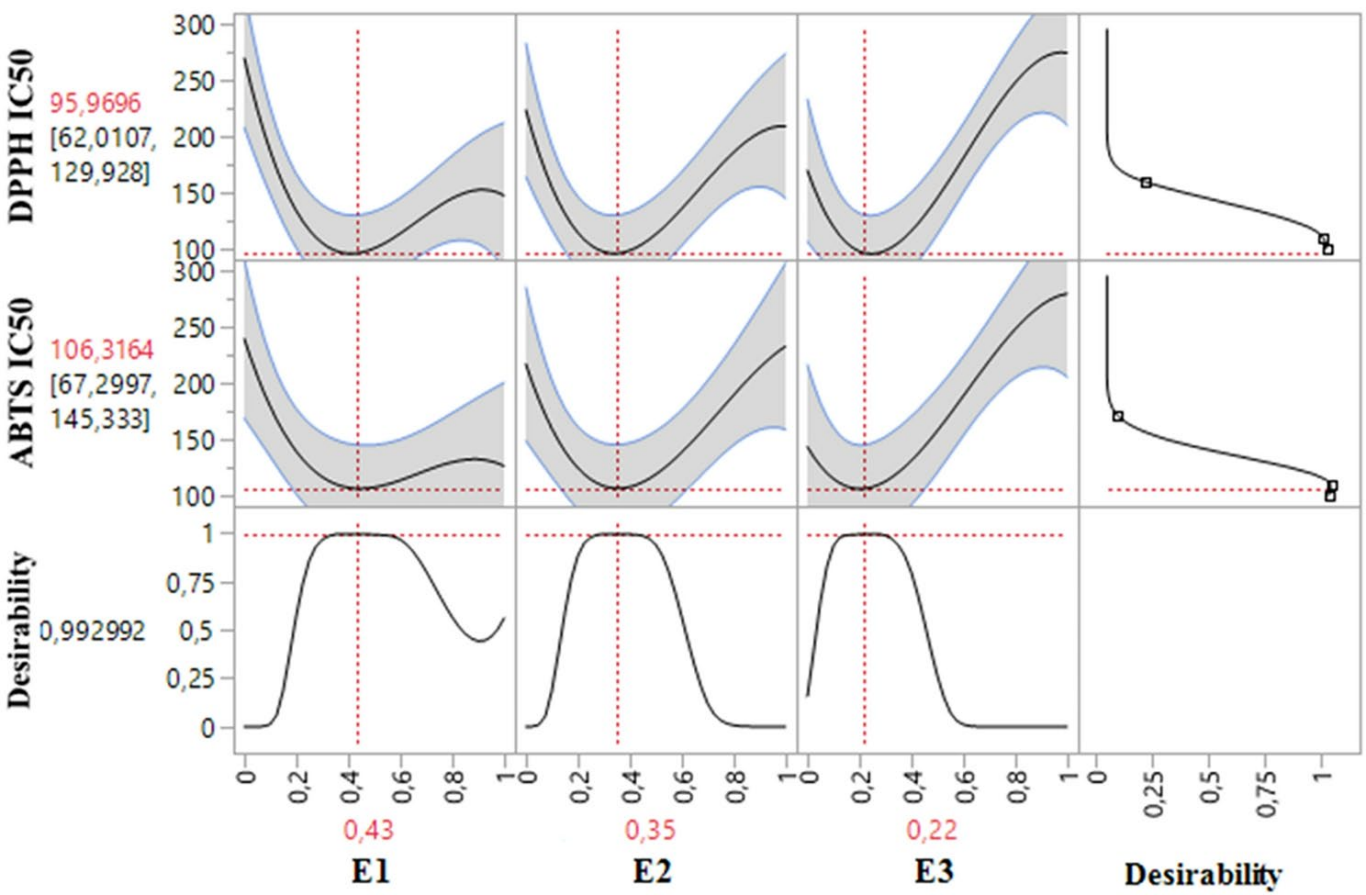
Desirability profile illustrates the precise proportions of E1, 32, and E3 extracts, leading to the optimum value for ABTS IC_50_

As illustrated in the 2D and 3D mixture diagrams (Fig. 6), the dark blue region represents the optimal compromise for achieving the best ABTS IC_50_ value of 116.76 µg/mL using a ternary mixture of E1, E2, and E3. This combination’s efficacy is validated by the desirability test (Fig. 7), which confirms that an optimal ABTSIC_50_ value of 116.76 µg/mL, with a desirability of 99%, can be achieved with the following proportions: 45% E1, 35% E2, and 20% E3. This level of antioxidant activity surpasses that of standard antioxidants such as BHT (IC_50_ = 127.15 ± 8.7 *µ*g/mL) and ascorbic acid (IC_50_ = 151.61 ± 7.45 *µ*g/mL).

### Simultaneous optimization of all responses

**Fig. 8:**
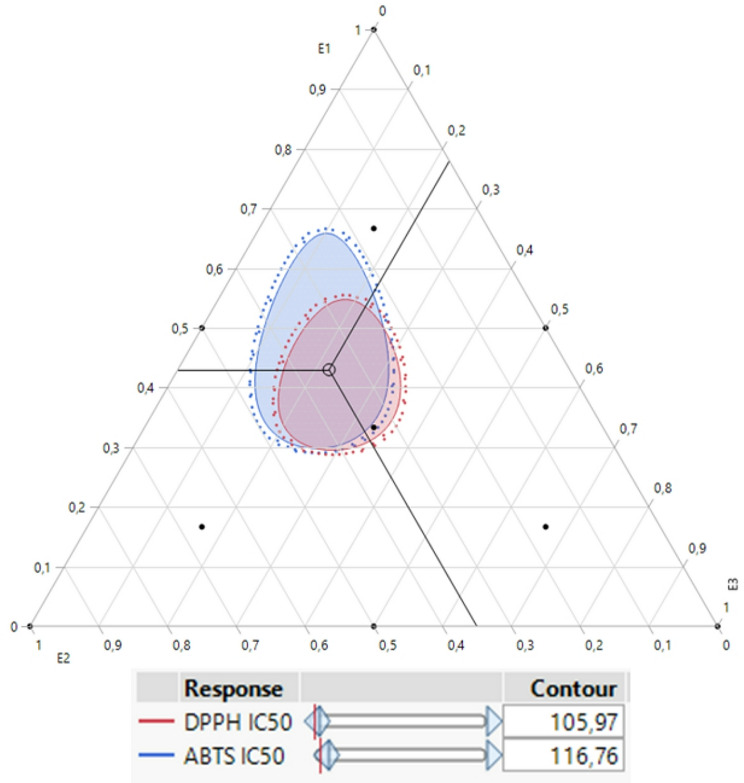
2D and 3D mixture plots of the desired compromise area, resulting in the best value DPPH IC_50_ (**A**), and ABTS IC_50_ (**B**)

**Fig. 9:**
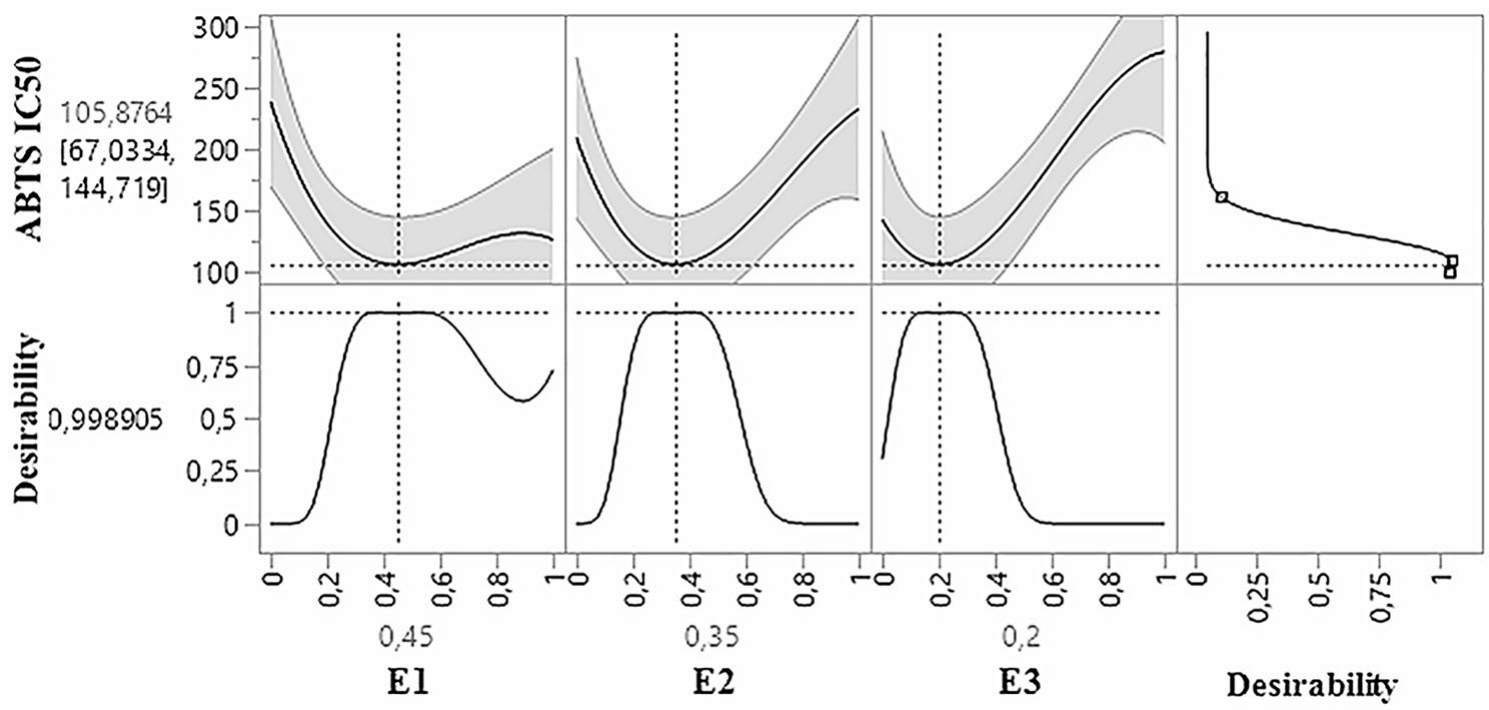
Desirability profile for simultaneous optimization showing the precise proportions of the three tested extracts, leading to optimal values for both responses (DPPH IC_50_ and ABTS IC_50_)

The desirability test not only accurately predicts DPPH IC_50_ and ABTS IC_50_ responses individually but also aids in identifying optimal conditions for their simultaneous optimization. In this study, the objective was to achieve an effective balance that enhances both responses concurrently. As illustrated in the desirability graphs (Figs. 8 and 9), we attained a remarkable optimization efficiency of 99.29% using a ternary blend consisting of 43% E1, 35% E2, and 22% E3 extracts. For this specific formulation, the optimal response values were 95.96 *µ*g/mL for DPPH IC_50_ and 106.31 *µ*g/mL for ABTS IC_50_.

### Experimental verification of the assumed model

The table presented offers a thorough validation of the cubic models used to assess the antioxidant effects of a blend of *Crocus sativus* extracts. This analysis is crucial to ensure the models accurately predict antioxidant activities, as measured by the DPPH_IC50_ and ABTS_IC50_ assays. The strong correlation between experimental results and predicted values highlights the model’s reliability and practical relevance.

In the specific results shown, the mixture consists of 43% CSSE, 35% CSLE, and 22% CSTE. The experimental DPPH IC_50_ value was recorded at 97.36 ± 1.23 *µ*g/mL, closely matching the predicted value of 95.66 ± 00.00 *µ*g/mL. Similarly, the experimental ABTS IC_50_ value of 110.59 ± 6.82 *µ*g/mL aligns with the predicted value of 106.31 ± 00.00 *µ*g/mL. These findings emphasize the model’s accuracy in predicting the antioxidant potential of these essential oil combinations under the tested conditions.

Validating these results is crucial, as it not only confirms the reliability of the modeling approach but also enhances our understanding of how different proportions of essential oils can synergistically boost antioxidant effects.

## Discussion

The study highlights the optimized formulation of antioxidant-rich extracts from *Crocus sativus* (stigmas, leaves, and tepals) through a simplex-centroid design approach, revealing their potential in mitigating oxidative stress. The ternary mixture demonstrated significant antioxidant capacity, as evidenced by low DPPH IC_50_ and ABTS IC_50_ values, outperforming synthetic antioxidants like butylated hydroxytoluene (BHT) and natural antioxidants such as ascorbic acid (AA). This aligns with prior findings emphasizing the high radical-scavenging activities of saffron carotenoids and phenolics, particularly crocin and rutin (Mashmoul et al. 2013; Rahaiee et al. 2015). Reactive oxygen species (ROS) are associated with numerous chronic diseases, such as aging, diabetes mellitus, cardiovascular diseases, cancer, and neurodegenerative disorders (Jomova et al. 2023). This connection is driven by the interplay between ROS generation and the propagation of cell signaling pathways, leading to extracellular matrix degradation and neurodegeneration (Ben Saad et al. 2023). Antioxidants, therefore, have emerged as essential preventive agents against these chronic diseases (Badraoui et al. 2025; Papandreou et al. 2011). The antioxidant properties of *Crocus sativus* have been extensively studied using assays such as DPPH, and ABTS.

The high antioxidant activity of stigmas, attributed to compounds such as crocin (11.04%) and ellagic acid (6%), is consistent with previous studies that identified these constituents as potent radical scavengers (Jomova et al. 2023). Similarly, the significant ellagic acid content in leaves (62.47%) and rutin in tepas (22.32%) suggests their poential as alternative antioxidant sources, corroborating the findings of Anbualakan et al. (2022) (Anbualakan et al. 2022), and (Singh et al. 2022). Unlike prior studies focusing solely on individual extracts (Rahaiee et al. 2015), this research underscores the synergistic effects of combining by-products, which exhibit improved antioxidant efficiency. This result is novel and substantiates the application of mixture design in optimizing plant extract formulations, as demonstrated in previous studies (Assaggaf et al. 2024; Elbouzidi et al. 2025).

The study’s use of simplex-centroid design to optimize the antioxidant activity of ternary mixtures represents an advancement over earlier research, where individual or binary combinations were analyzed. Mixtures such as 43% stigmas, 35% leaves, and 22% tepals exhibited a desirability of 99.29%, achieving optimal antioxidant effects. Similar approaches have been adopted in essential oil research for maximizing antioxidant and antimicrobial activities (Assaggaf et al. 2024; Elbouzidi et al. 2025), but their application to *Crocus sativus* parts is pioneering. The statistical validation of the cubic model, with high R² values (0.93 for DPPH IC_50_ and 0.91 for ABTS IC_50_), emphasizes the reliability of this approach. This contrasts with earlier studies that lacked comprehensive modeling to predict antioxidant responses.

This study contributes significantly to the valorization of *Crocus sativus* by-products, which are often underutilized. Previous research (Ben-Othman et al. 2020), highlighted the economic and environmental benefits of repurposing agricultural waste into bioactive formulations. By optimizing stigmas, leaves, and tepals, this study aligns with global sustainability goals, reducing waste while enhancing the commercial value of saffron cultivation. While crocin and other carotenoids in stigmas have been extensively documented (Rahaiee et al. 2015), this study is among the first to explore the synergistic antioxidant potential of *Crocus sativus* leaves and tepals in combination with stigmas. The significant enhancement of antioxidant activity in ternary mixtures positions these formulations as viable alternatives to synthetic antioxidants, with applications in food preservation, cosmetics, and nutraceuticals.

Although this study provides promising results regarding the synergistic antioxidant potential of the stigmas, leaves, and tepals of *Crocus sativus* L., certain limitations should be acknowledged. First, the experiments were conducted exclusively in vitro, which may not fully reflect the complexity of in vivo biological interactions. Further studies, including animal or clinical models, are needed to assess the bioavailability and physiological effects of the optimized mixtures.

Second, the hydroethanolic extraction method employed may not recover the full spectrum of bioactive compounds present in the plant matrices. The use of alternative extraction techniques (e.g., supercritical fluid extraction, aqueous extraction) could yield different phytochemical profiles and antioxidant activities, thus warranting further investigation.

## Conclusions

This study successfully optimized a three-component extract mixture derived from *Crocus sativus* L. stigmas, leaves, and tepals, demonstrating their significant antioxidant potential. Using a simplex-centroid design, the study identified optimal ternary combinations for DPPH and ABTS assays, achieving IC_50_ values of 95.96 *µ*g/mL and 106.31 *µ*g/mL, respectively. These results surpass the antioxidant activities of standard synthetic and natural antioxidants, such as butylated hydroxytoluene and ascorbic acid. Chemical profiling revealed that the extracts are rich in bioactive compounds, including crocin, rutin, and ellagic acid, which contribute to their antioxidant efficacy. By valorizing saffron by-products, often discarded as agricultural waste, this research provides a sustainable and economically viable approach to improving saffron cultivation while expanding applications in food preservation, nutraceuticals, and biopharmaceuticals.

Future research should focus on validating the antioxidant effects of these mixtures through in vivo studies and exploring their potential applications in functional foods, pharmaceuticals, and cosmetics. Investigating the molecular mechanisms underlying the synergistic interactions of bioactive compounds could provide deeper insights into their efficacy. Additionally, studies on the scalability and sustainability of extraction processes, as well as bioavailability and stability of the mixtures, are crucial for practical applications. These efforts will further enhance the utilization of saffron by-products, contributing to sustainable agriculture and innovative healthcare solutions.

## Electronic supplementary material

Below is the link to the electronic supplementary material.

Supplementary Material 1: The following supporting information can be downloaded at: Figure S1: title; TIC chromatogram, at a wavelength of 254 nm of hydroethanolic extract of C. sativus leaves. Figure S2: TIC chromatogram, at a wavelength of 254 nm of hydroethanolic extract of C. sativus stigmas. Figure S3: TIC chromatogram, at a wavelength of 254 nm of hydroethanolic extract of *C. sativus* tepals. Table S1: Phytochemical profile of the extracts using HPLC-DAD.

## Data Availability

The datasets generated and analyzed during the current study are available from the corresponding author upon request.
