# Supplementary material for: Optimized formulation of a three-component extract mixture from Moroccan Crocus sativus L. (Stigmas, leaves, and Tepals) for enhanced antioxidant activity
Source: Bioresour Bioprocess. 2025 Sep 1;12(1):94. doi: 10.1186/s40643-025-00892-7 (PMC12401861; doi:10.1186/s40643-025-00892-7)
Supplement: Supplementary file 1 — Supplementary Material 1: The following supporting information can be downloaded at: Figure S1: title; TIC chromatogram, at a wavelength of 254 nm of hydroethanolic extract of C. sativus leaves. Figure S2: TIC chromatogram, at a wavelength of 254 nm of hydroethanolic extract of C. sativus stigmas. Figure S3: TIC chromatogram, at a wavelength of 254 nm of hydroethanolic extract of C. sativus tepals. Table S1: Phytochemical profile of the extracts using HPLC-DAD. [file 40643_2025_892_MOESM1_ESM.docx]

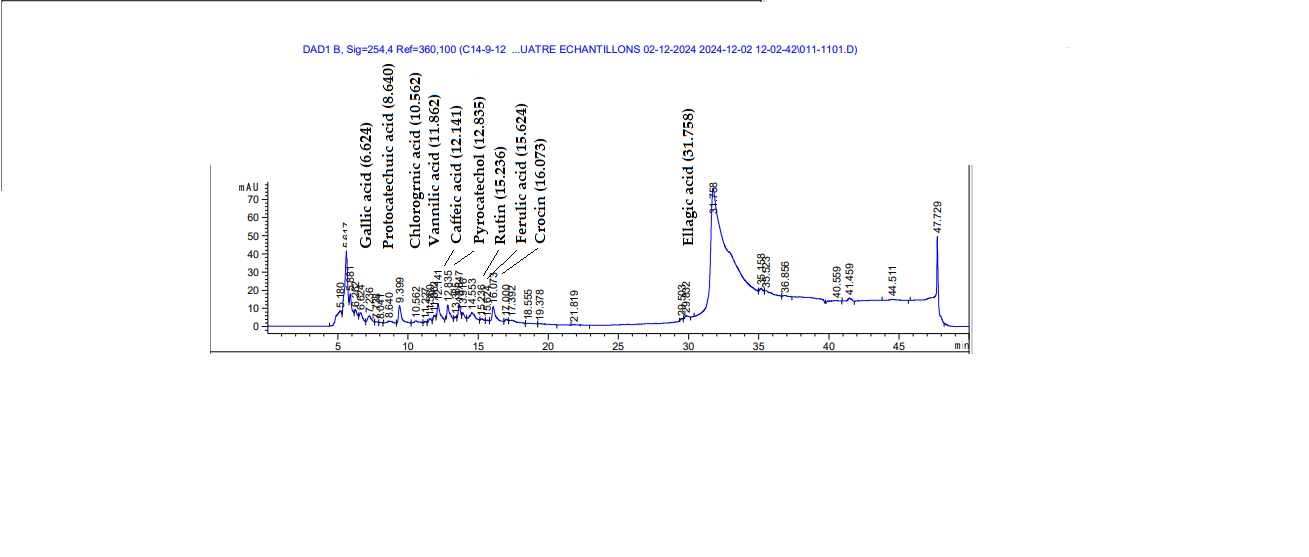


**Figure S1.** TIC chromatogram, at a wavelength of 254 nm of hydroethanolic extract of *C. sativus* leaves.


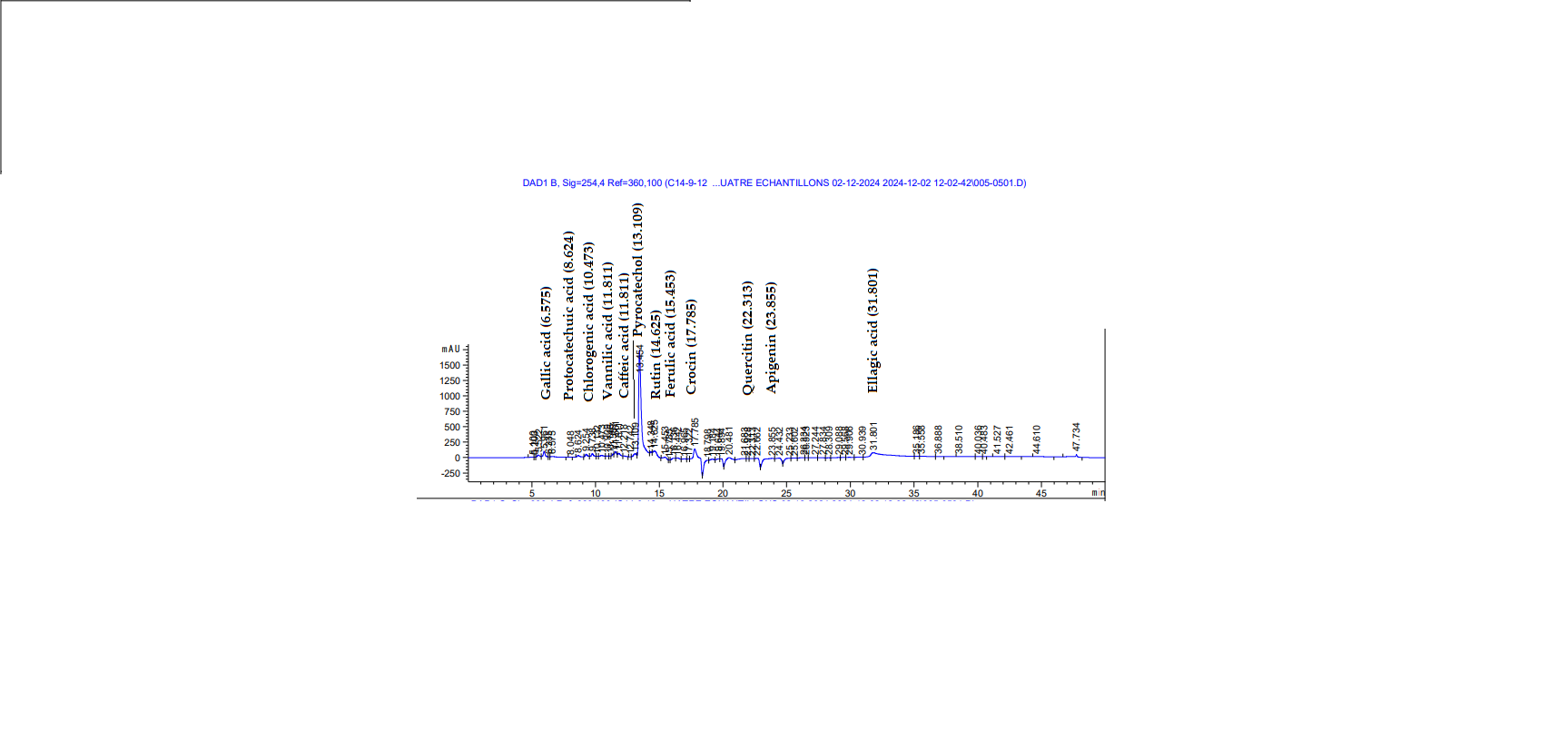


**Figure S2.** TIC chromatogram, at a wavelength of 254 nm of hydroethanolic extract of *C. sativus* stigmas.


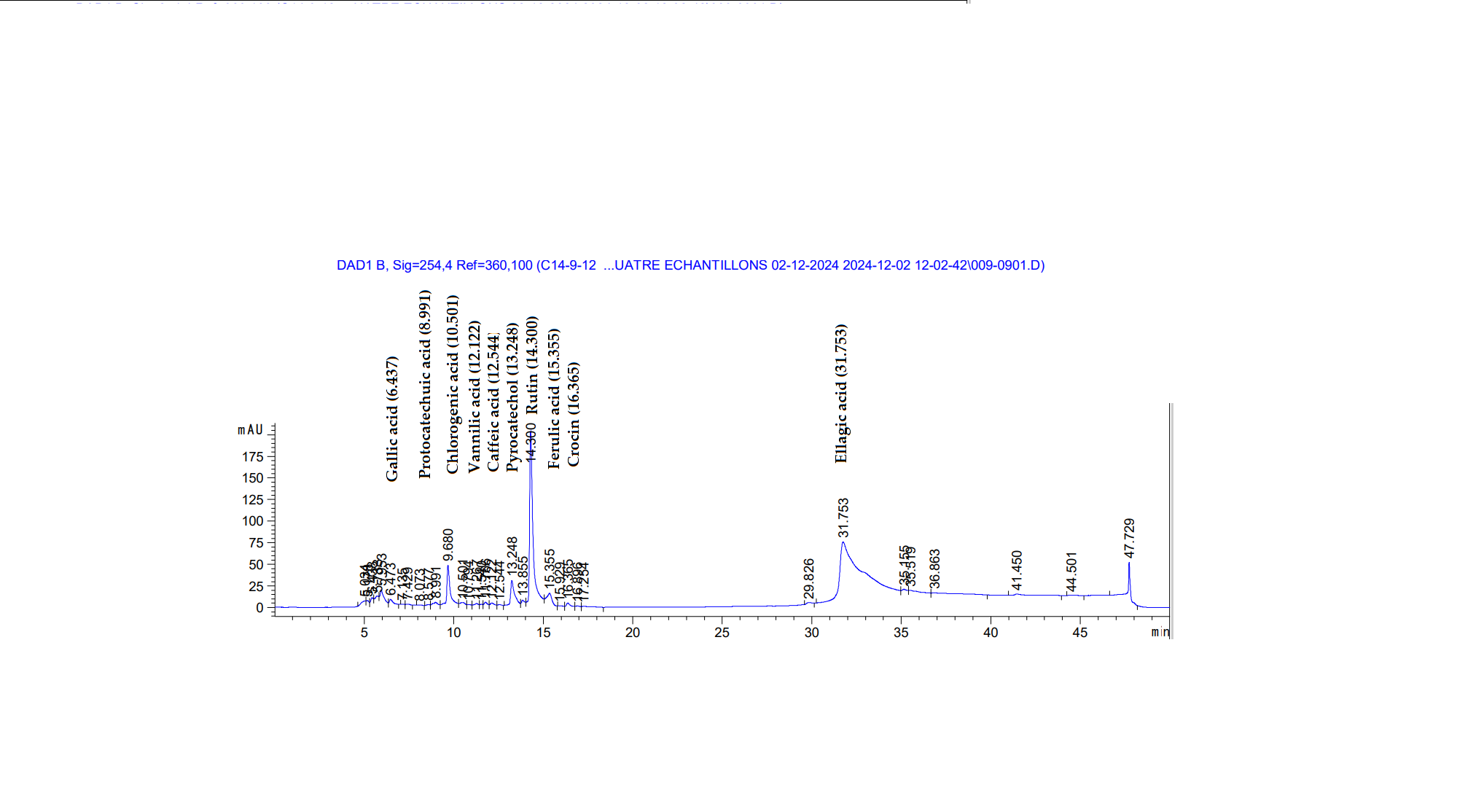


**Figure S3.** TIC chromatogram, at a wavelength of 254 nm of hydroethanolic extract of *C. sativus* tepals.

**Table S1.** Phytochemical profile of the extracts using HPLC-DAD.

| **Peak No.** | **Extract RT (min)** | **Closest Standard RT (min)** | **Compound** | **Area (mAU*s)** | **Area (%)** |
| --- | --- | --- | --- | --- | --- |
| **CSLE** | | | | | |
| **5** | 6.624 | Gallic acid (6.41) | Gallic acid | 82.89214 | 0.8712 |
| **9** | 8.640 | Protocatechuic acid (8.79) | Protocatechuic acid | 22.17080 | 0.2330 |
| **11** | 10.562 | Chlorogenic acid (10.60) | Chlorogenic acid | 21.32190 | 0.2241 |
| **14** | 11.862 | Vanillic acid (12.16) | Vanillic acid | 30.85578 | 0.3243 |
| **15** | 12.141 | Caffeic acid (12.20) | Caffeic acid | 121.59741 | 1.2780 |
| **16** | 12.835 | Pyrocatechol (12.71/13.29) | Pyrocatechol | 93.04482 | 0.9779 |
| **21** | 15.236 | Rutin (14.89) | Rutin | 9.47628 | 0.0996 |
| **22** | 15.624 | Ferulic acid (15.77) | Ferulic acid | 2.84809 | 0.0299 |
| **23** | 16.073 | Crocin (16.47) | Crocin | 126.32553 | 1.3277 |
| **31** | **31.758** | **Ellagic acid (31.73)** | **Ellagic acid** | **5943.23193** | **62.4659** |
| **33** | 35.523 | - | Unknown | 175.92670 | 1.8491 |
|  |  |  | **Total Area (%)** | **67.8316** | |
| **CSSE** | | | | | |
| **6** | 6.575 | Gallic acid (6.41) | Gallic acid | 226.22218 | 0.1936 |
| **8** | 8.624 | Protocatechuic acid (8.79) | Protocatechuic acid | 298.78717 | 0.2557 |
| **12** | 10.473 | Chlorogenic acid (10.60) | Chlorogenic acid | 249.93330 | 0.2139 |
| **17** | 11.811 | Vanillic acid (12.16) | Vanillic acid | 532.99579 | 0.4561 |
| **18** | 12.218 | Caffeic acid (12.20) | Caffeic acid | 54.94732 | 0.0470 |
| **20** | 13.109 | Pyrocatechol (12.71) | Pyrocatechol | 780.20245 | 0.6676 |
| **23** | 14.625 | Rutin (14.89) | Rutin | 2387.34033 | 2.0428 |
| **24** | 15.453 | Ferulic acid (15.77) | Ferulic acid | 519.75189 | 0.4447 |
| **30** | 17.785 | Crocin (16.47) | Crocin | 1.29011e4 | 11.0392 |
| **38** | 22.313 | Quercetin (22.30) | Quercetin | 3346.86450 | 2.8638 |
| **40** | 23.855 | Apigenin (23.62) | Apigenin | 6752.21240 | 5.7777 |
| **53** | 31.801 | **Ellagic acid (31.73)** | **Ellagic acid** | **7016.58154** | **6.0039** |
|  |  |  | **Total Area (%)** | **30.006** | |
| **CSTE** | | | | | |
| **6** | 6.473 | Gallic acid (6.41) | Gallic acid | 46.38789 | 0.3919 |
| **11** | 8.991 | Protocatechuic acid (8.79) | Protocatechuic acid | 57.63250 | 0.4869 |
| **13** | 10.501 | Chlorogenic acid (10.60) | Chlorogenic acid | 47.64726 | 0.4025 |
| **18** | 12.122 | Vanillic acid (12.16) | Vanillic acid | 29.95723 | 0.2531 |
| **19** | 12.544 | Caffeic acid (12.20) | Caffeic acid | 7.58499 | 0.0641 |
| **20** | 13.248 | Pyrocatechol (13.29) | Pyrocatechol | 388.49808 | 3.2820 |
| **22** | 14.300 | Rutin (14.89) | Rutin | 2642.38330 | 22.3230 |
| **23** | 15.355 | Ferulic acid (15.77) | Ferulic acid | 310.23038 | 2.6208 |
| **25** | 16.365 | Crocin | Crocin | 50.11451 | 0.4234 |
| **29** | 31.753 | **Ellagic acid (31.73)** | **Ellagic acid** | **5975.12646** | **50.4781** |
|  |  |  | **Total Area (%)** | **80.7258** | |
